# Activation of S1P_2_ is protective against cisplatin‐induced peripheral neuropathy

**DOI:** 10.1111/cpr.13549

**Published:** 2023-09-19

**Authors:** Brenda Wan Shing Lam, Ping Xiang, Boya Peng, Ling Jun Joshua Soon, Amelia Ting Yu Yam, Claudine Ming Hui Lim, Yu Zheng, Long N. Nguyen, Deron R. Herr, Minh T. N. Le

**Affiliations:** ^1^ Department of Pharmacology and Institute for Digital Medicine, Yong Loo Lin School of Medicine National University of Singapore Singapore Singapore; ^2^ Department of Surgery, Yong Loo Lin School of Medicine National University of Singapore Singapore Singapore; ^3^ Institute of Molecular and Cell Biology Agency for Science, Technology and Research (A*STAR) Singapore Singapore; ^4^ Department of Physiology, Yong Loo Lin School of Medicine National University of Singapore Singapore Singapore; ^5^ Department of Biochemistry, Yong Loo Lin School of Medicine National University of Singapore Singapore Singapore; ^6^ Singapore Lipidomics Incubator (SLING), Life Sciences Institute National University of Singapore Singapore Singapore; ^7^ Cardiovascular Disease Research (CVD) Programme, Yong Loo Lin School of Medicine National University of Singapore Singapore Singapore; ^8^ Immunology Translational Research Program, Yong Loo Lin School of Medicine National University of Singapore Singapore Singapore; ^9^ Translational Neuroscience Initiative Sanford Burnham Prebys Medical Discovery Institute La Jolla California USA

Dear Editor,

Platinum‐based therapeutics are commonly used for cancer treatment, but often lead to peripheral neuropathy. Peripheral neuropathy is characterised by nerve damage in the peripheral nervous system (PNS). It can cause various sensory alterations, such as paresthesia, allodynia, or hyperalgesia, and significantly impact patients' quality of life.^1^ Multiple factors, including chemotherapy, diabetes, and autoimmunity, contribute to peripheral neuropathy.^2^ Chemotherapy‐induced peripheral neuropathy (CIPN) affects a substantial number of cancer patients even after the treatment completion. In a post‐chemotherapy follow‐up study, it was observed that up to 30% of the patients continued to suffer from CIPN.^3^ Despite information regarding molecular events underlying peripheral neuropathy, there are no effective treatments available to prevent or reverse CIPN, consequently resulting in dose reduction or cessation of chemotherapy, which may compromise patient survival. Currently, the main focus is on prevention and alleviating symptoms, while clinical trials addressing the underlying mechanisms of the disorder continue to be limited. For example, a phase II study in 2012 investigated the effectiveness of tetrodotoxin for pain relief related to chemotherapy (NCT01655823), and a phase III study probed the neuroprotective effects of amifostine in treating peripheral neuropathy (NCT00058071). However, there have been no significant updates on the progress of these two drugs since then, highlighting the gap in our understanding of the disease's mechanisms and emphasising the urgent need for further research to develop viable treatment options for this condition. This underscores the importance of identifying therapeutic targets, such as the S1P_2_ receptor, which presents promising avenues for developing interventions to mitigate or prevent the neurotoxic effects of platinum‐based chemotherapy drugs.

Building on our previous research, we have discovered that activating the S1P_2_ receptor can counteract the behavioural changes, myelin defects, and satellite glial cell activation in the dorsal root ganglia (DRG) induced by cisplatin, a platinum‐based chemotherapy drug. In this study, we focus on cisplatin and investigate the mechanistic basis for the neuroprotective effects of activating the S1P_2_ receptor. The results demonstrate that cisplatin treatment in vivo leads to significant alterations in major neuronal‐associated pathways within the DRG, a site of peripheral nerve damage. Importantly, co‐treatment with CYM‐5478, an S1P_2_ activator, restores these pathway changes. These findings highlight the potential of targeting the S1P_2_ receptor as a pharmacological approach for rescuing CIPN.

## MATERIALS AND METHODS

### Animals and drug treatment

Twelve female Sprague–Dawley (S.D.) rats (InVivos, Singapore) were used with four rats per treatment group. The experimental procedures were approved by the Institutional Animal Care and Use Committee at the National University of Singapore.

**FIGURE 1:**
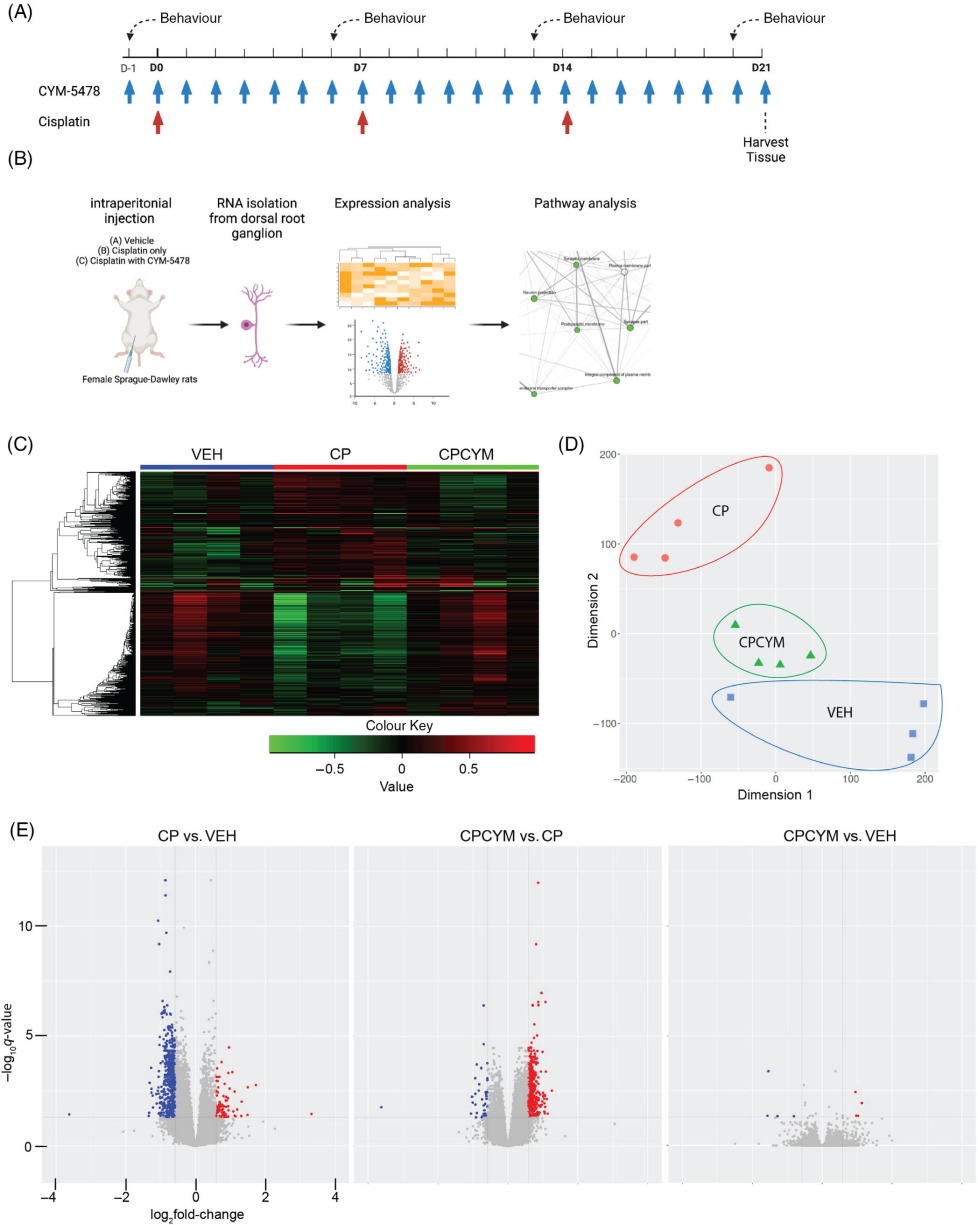
S1P_2_ activation rescued transcriptomic changes induced by cisplatin treatment. (A) Graphical depiction of the in vivo treatment. (B) Graphical depiction of the RNA‐Seq workflow to explore the relationship between samples. (C) Differential expression analysis was performed to obtain differentially expressed genes with a fold change cut‐off of ±1.5 and *q*‐value <0.05, depicted as a heat map. Upregulated genes are depicted in red, while downregulated genes are depicted in green. (D) t‐distributed stochastic neighbour embedding (t‐SNE) projection was used to visualise clustering of the RNASeq gene sets. Blue represents vehicle‐treated group. Red represents cisplatin‐treated group. Green represents cisplatin and CYM‐5478 co‐administered group. (E) Volcano plots were used to highlight the statistical significance and magnitude of change of RNASeq between two treatment groups. Red dots represent genes, which were significantly upregulated (*q*‐value <0.05, fold change ≥1.5). Blue dots represent genes, which were significantly downregulated (*q*‐value <0.05, fold change ≤−1.5). CP, samples from cisplatin‐treated rats; CPCYM, samples from rats receiving co‐administration of cisplatin and CYM‐5478; VEH, samples from vehicle‐treated rats.

Cisplatin (Sigma, USA) was dissolved in sterile saline prior to each use. The treatment, starting on Day −1 (Figure 1A), followed a dosage previously shown to induce peripheral neuropathy in rats.^4^

### RNA‐sequencing and analysis

RNA was isolated from rat DRG using RNeasy Micro Kit (QIAGEN, USA) and analysed with NanoDrop (ThermoScientific, USA). The RNA‐sequencing library was prepared with rRNA‐depleted RNA by NEBNext Ultra Directional RNA Library Prep Kit for Illumina (NEB, USA). Clustering of the index‐coded samples was performed on a cBot Cluster Generation System using PE Cluster Kit cBot‐HS (Illumina, USA). Raw base call files from Bowtie v2.0.6 were demultiplexed with bcl2fastq v2.20, and FastQC was used for quality control. Alignment to Rnor_6.0 (*Rattus norvegicus*, Norway rat, genome) was done with STAR‐2.7.8a and read counts were quantified with ‐quantMode GeneCounts enabled. Gene read count tab‐delimited files (TSV) were consolidated into a comma‐delimited file (CSV) with Modern CSV v1.3.23. iDep0.91 and DESeq2 were used for differential expression analysis, considering genes with a fold change of ±1.5 (*q*‐value <0.05) as differentially expressed genes (DEGs). Functional analysis of DEGs utilised Gene ontology (GO) terms and Kyoto Encyclopedia of Genes and Genomes (KEGG) terms through ShinyGO v0.61 (*q*‐value <0.05). Gene set enrichment analysis (GSEA) was performed with the GSEA software (Broad Institute), and gene symbols were converted from rat to human using the ‘Rat_Gene_Symbol_Remapping_Human_Orthologs_MSigDB.v7.2.chip’. Gene set permutations were performed 100,000 times to determine *p*‐values.

### Primary DRG neuronal cell isolation and treatment

Primary DRG neurons were isolated from 4‐ to 6‐week‐old C57BL/6 female mice (InVivos) as previously described.^5^ Cisplatin (40 mM; Sigma) was dissolved in dimethylformamide (Sigma) prior to each use. CYM‐5478 (1 mM) was dissolved in fatty acid‐free fetal bovine serum (Sigma).

### Immunofluorescence

Primary DRG neuronal cells were fixed in 4% ice‐cold paraformaldehyde (Sigma) overnight. Cells were blocked with 2.5% bovine serum albumin (Biowest, France) and 0.1% Triton‐X‐100 (Sigma) for 1 h, followed by overnight incubation with anti‐beta‐tubulin antibody (Tuj1; R&D Systems, MAB1195) at 25 μg/mL in blocking buffer at 4°C. Cells were washed three times with 1× PBS (ThermoScientific) before incubation with goat anti‐mouse IgG Alexa Fluor 488‐conjugated secondary antibody (Jackson ImmunoResearch, USA) diluted 1:250 in blocking buffer for 2 h. Cells were counter‐stained with Hoechst 33342 (ThermoScientific) at 1:500, and washed three times in 1× PBS. The slides were mounted with anti‐fade mounting media (Vector Laboratories, USA). Images were captured at 40× magnification with an epifluorescence microscope (Leica DM 6B) and scored for neurite growth using ImageJ by a blinded researcher.

### Statistical analysis

An ANOVA with Tukey's multiple comparison test in GraphPad Prism version 8 was used to determine the significance of differences between the treated samples and controls for values resulted from immunofluorescence. A *p*‐value <0.05 was considered significant based on at least three independent replicates. For DGE and GO analysis, the *q*‐value of any enrichment was calculated with DESeq2 within iDep0.91.

## RESULTS

### S1P_2_ activation rescued molecular pathology associated with cisplatin treatment in vivo

To determine the pathways underlying the protective effect of S1P_2_ on CIPN, DRGs were collected from rats treated with cisplatin alone or co‐treated with CYM‐5478 (Figure 1A). RNA from the DRGs was extracted, purified, and subjected to RNASeq analysis (Figure 1B). The RNA samples were of high quality, free of contamination, and passed quality assessment by Novogene.

The transcriptomic patterns of DRGs collected from CP rats differed from VEH rats, while co‐treatment with CYM‐5478 (CPCYM) resulted in transcriptomic patterns resembling those of VEH rats (Figure 1C). To further validate the variation in the expression pattern between CPCYM and CP rats, we performed t‐SNE analysis. Overall, the expression profile of DRGs from CPCYM and VEH rats were more closely clustered to each other as compared to CP rats (Figure 1D).

Out of the 660 genes that were meaningfully (fold change ≥2) and significantly (*q*‐value <0.05) differentially expressed in DRGs of rats following CP treatment, 581 genes were upregulated, and 79 genes were downregulated (Figure 1E). Notably, co‐administration of cisplatin with CYM‐5478 reduced the differences in gene expression, with only nine genes showing meaningfully (fold change ≥2) and significantly (*q*‐value <0.05) differential expression (Figure 1E). Among the top downregulated genes, several were associated with mitochondrial function (*ckmt2* and *ckmt1b*) and neurite growth (*csmd3*, *cdh4*, and *slitrk4*), both of which are relevant to CIPN.^6^, ^7^, ^8^ Collectively, these data suggest that S1P_2_ activation in DRGs can restore cisplatin‐induced gene dysregulation associated with mitochondrial functions and neurite growth.

### S1P_2_ activation restored dysregulated neuronal‐associated pathways in cisplatin‐treated rats

**FIGURE 2:**
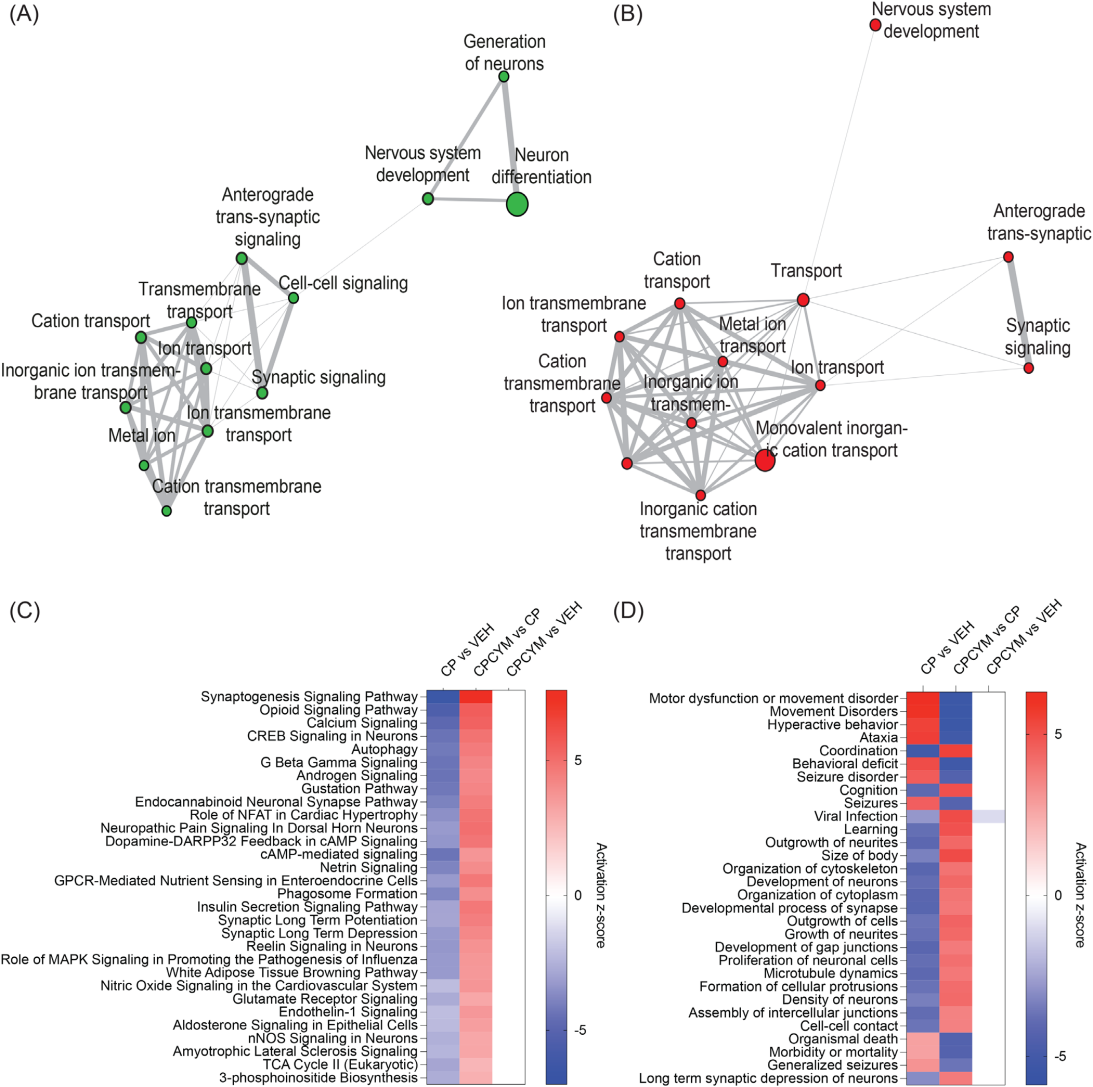
S1P_2_ activation restored dysregulated neuronal‐associated pathways in cisplatin‐treated rats. Gene ontology analysis for canonical biological processes was carried out based on the differential gene analysis and illustrated using network. (A) Comparison between cisplatin versus vehicle‐treated rats. (B) Comparison between cisplatin and CYM‐5478. Red represents more significantly enriched. Green represents less significantly enriched (fold change ≥1.5, *q*‐value <0.05). The larger circle in each plot represents most significantly enriched. (C) Ingenuity pathway analysis was carried out based on the differential gene analysis. The top 30 canonical pathways, which are significantly changed (−2 ≤ *z*‐score ≥ 2) in cisplatin‐treated rats, are illustrated as heatmap. (D) The top 30 diseases/functions associated pathways which are significantly changed (−2 ≤ *z*‐score ≥ 2) in cisplatin‐treated rats are also illustrated as heatmap. CP, cisplatin; CPCYM, co‐administration of cisplatin with CYM‐5478; VEH, control rat.

The GSEA for up‐ and down‐regulated genes were also performed for Gene Ontology (GO) terms for biological process. Cisplatin‐treated rats revealed a significant downregulation of synaptic function‐related processes, including anterograde trans‐synaptic signalling (*q*‐value = 9.85E−38), synaptic signalling (*q*‐value = 9.85E−38), and cell–cell signalling (*q*‐value = 3.91E−27) (Figure 2A). We also observed significant downregulation of processes associated with ion transports, such as ion transmembrane transport (*q*‐value = 2.02E−30), ion transport (*q*‐value = 6.06E−29), inorganic transmembrane transport (*q*‐value = 4.38E−28), cation transport (*q*‐value = 5.51E−26), and metal ion transport (*q*‐value = 5.51E−26). However, co‐administration CYM‐5478 reversed these effects and resulted in upregulation of these processes (Figure 2B).

We used IPA to identify canonical pathways, and disease function annotations associated with the differentially regulated genes with *q*‐value <0.05. Consistent with the GO analysis, synaptogenesis signalling pathway, related to synaptic function, was the most inactivated biological process in DRGs of cisplatin‐treated rats (Figure 2C). Calcium signalling, known for its important roles in maintaining neuronal functions,^9^, ^10^, ^11^ was activated in DRGs following cisplatin treatment. Interestingly, the pathway associated with neuropathic pain signalling in dorsal horn neurons was significantly activated in cisplatin‐treated rats. Notably, all these dysregulated pathways were corrected with co‐administration of CYM‐5478.

The DEGs were also sorted into related diseases and functions using IPA and triaged into the top 30 categories (Figure 2D). Remarkably, the top three significantly altered pathways in cisplatin‐treated rats were associated with movement disorders. Also, we observed an increased activation of pathways associated with changes in neuronal cytoskeleton structures, such as outgrowth of neurites, body size, and cytoskeleton organisation. These findings suggest that cisplatin treatment damages neurons, leading to possible compensatory activation of these pathways. Notably, co‐administration of CYM‐5478 rescued these dysregulated categories associated with diseases and functions.

The results of these two independent analyses indicate that S1P_2_ activation has a protective effect against cisplatin‐induced neuronal defects in the DRGs.

### S1P_2_ activation reduced cisplatin‐induced neurite damage in rat DRG and primary murine DRG neurons

**FIGURE 3:**
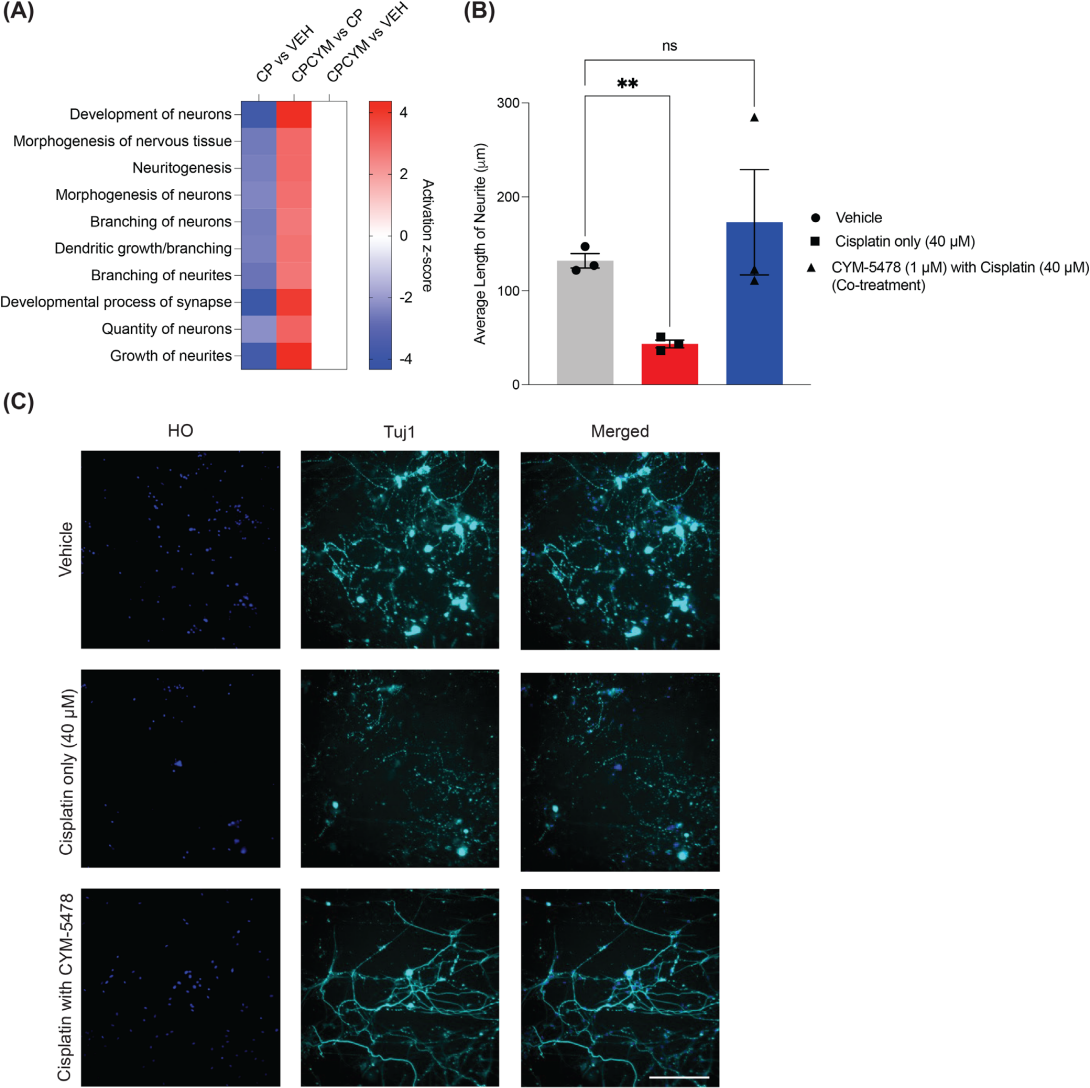
S1P_2_ activation reduced cisplatin‐induced neurite damage in rat dorsal root ganglia (DRG) and primary mouse DRG neurons. (A) The top 10 neurite‐associated pathways by IPA, which changed (−2 ≤ *z*‐score ≥ 2) in cisplatin‐treated rats. (B, C) Primary DRG neurons were isolated from 4‐ to 6‐week‐old C57BL/6 mice and cultured for 5 days before treatment. Cells were fixed and stained with Hoechst 33342 (HO) and anti‐beta‐tubulin‐III (Tuj1). The length of neurites was quantified using ImageJ from *n* = 3 mice per group. scale bar: 200 μm. **p < 0.001. Error bars represent standard error of mean.

Due to the known role of axonal degeneration in the development of CIPN, IPA terms specific to neurite integrity were evaluated (Figure 3A).^12^ The results supported the notion that cisplatin induced neurite damage as seen from excessive activation of pathways associated with neuritogenesis, morphogenesis of neurons, and branching of neurites (Figure 3A). To confirm this, primary C57BL/6 mice DRG neurons were examined using immunofluorescent staining of the neuronal markers beta‐tubulin‐III (Tuj1). It was observed that cisplatin treatment significantly reduced neurite length (Figure 3B,C). However, co‐administration of CYM‐5478 fully restored neurite length, indicating that S1P_2_ activation can reduce cisplatin‐induced neurite damage.

## DISCUSSION

This study aimed to further our understanding of the mechanisms underlying the neuroprotective effect of S1P_2_ in CIPN. CIPN is a common side effect of platinum‐based chemotherapy, and currently, there are no approved methods to mitigate this adverse effect other than discontinuing treatment. Previous study from the Herr lab demonstrated that S1P_2_ activation can prevent CIPN.^13^ The current study employs next‐generation sequencing to understand the molecular changes occurring in the DRG following cisplatin treatment, and to identify the mechanisms by which S1P_2_ activation exerts its protective effect.

Our in vivo findings suggest that cisplatin treatment in rats disrupted gene expression patterns related to neuronal growth and survival. This aligns with previous studies that used animal models of CIPN, which demonstrate cell death in the hippocampus and significantly greater DRG neuronal cell death with cisplatin compared to oxaliplatin, a less neuropathic platinum chemotherapeutic.^14^, ^15^ They attributed this difference in cell death as a result of greater platinum accumulation in neuronal cells treated with cisplatin. For example, cisplatin treatment led to death of primary cortical neurons in a dose‐dependent manner.^16^ We also observed that these disrupted pathways could be restored by treatment with CYM‐5478, an activator of S1P_2_.

Furthermore, S1P_2_ activation appears to have a rescue effect on cisplatin‐induced neuronal defects. We found that CIPN rat model exhibited a significant decrease in genes associated with various neuronal pathways, such as neuronal differentiation, generation of neurons, and synaptic signalling. However, co‐treatment with CYM‐5478 rescued the gene expression profiles of these pathways. In addition, analysis using the IPA revealed a decrease in the activation of neuronal pathways, including synaptogenesis signalling pathway, cAMP response element‐binding (CREB) signalling in neurons, and endocannabinoid neuronal synapse pathway. These data suggest that the impact of cisplatin on synaptogenesis signalling pathway, which has the lowest *z*‐score, might be related to its binding to neuronal tubulins, potentially affecting cell‐to‐cell communication in the PNS. Further studies could explore these effects and how S1P_2_ activation helps rescue them.

As shown in our previous data, S1P_2_ activation reduced gliosis in satellite glial cells (SGCs), which resemble astrocytes in the CNS.^13^ SGC and astrocytes activation are indicators of nervous system damage.^17^, ^18^ It has been suggested that aberrant electrical activity in neurons can induce the production of nitric oxide which activates SGCs, possibly through the extracellular signal‐regulated kinase (ERK) signalling pathway.^18^, ^19^ Activated SGCs release inflammatory cytokines which can cause damage to neuronal cells and form a positive feedback loop.^18^ Thus, changes in SGCs and neuronal cells induced by cisplatin may be a result of S1P_2_ activation.

The ability of CYM‐5478 treatment to correct pathways disrupted by cisplatin administration suggests that S1P_2_ activation acts against the molecular pathologies responsible for the disease. Importantly, this suggests that S1P_2_ agonists represent potentially disease‐modifying therapeutics rather than merely palliative treatments. Our previous work demonstrated that CYM‐5478 can attenuate cisplatin‐induced generation of reactive oxygen species (ROS) mediated by Rac1‐induced NADPH oxidase (NOX) activation.^20^ Since NOX‐mediated oxidative stress is a key driver of neuropathy,^21^ preventing this proximal event through S1P_2_ activation would be expected to prevent downstream pathway dysregulation.

Neurites, projections from neuronal cell bodies, play a crucial role in facilitating proper connectivity between cells in the CNS.^22^ Disrupted neurite outgrowth is associated with various neurodegenerative diseases, such as Alzheimer's and Parkinson's.^23^, ^24^, ^25^, ^26^ Several studies have explored regulating neurite outgrowth as a therapeutic approach for peripheral neuropathy. For example, guaifenesin improves neurite outgrowth in cultured DRG neurons,^27^ while donepezil reduces nerve degeneration in rats possibly by regulating neurite growth.^28^ Rat adipose‐derived stem cells have also been evaluated for peripheral neuropathy treatment based on neurite outgrowth.^29^ In murine DRG neurons, we observed that exposure to cisplatin led to reduction in neurite length, but co‐administration with CYM‐5478 rescued this damage. Interestingly, S1P_2_ activates Rho/Rho‐associated kinase pathway (ROCK), known to promote neurite retraction, while inhibition of ROCK has been shown to promote neurite outgrowth.^30^, ^31^, ^32^ This suggests that the neuronal protective effect of S1P_2_ may involve an indirect pathway, such as reduction in ROS. Inhibiting NOX promotes neurite growth, and activating S1P_2_ can suppress NOX.^20^, ^33^ Therefore, the neuroprotective effect of S1P_2_ activation may be attributed to inhibiting ROS formation through the RhoA/NOX3 pathway.^34^

In conclusion, the findings of this study suggest that S1P_2_ activation can rescue dysregulated genes and pathways associated with neuronal growth and survival, and attenuate neurite damage in cisplatin‐treated DRGs. To gain a more precise understanding of how S1P_2_ activation operates, conducting RNASeq at an earlier time point could help examine the direct pathways. Additionally, it would be interesting to investigate whether these pathways are specific to certain cell types or consistent across various tissues. Additional in vitro research could shed light on the significance of S1P_2_ activation in the recovery of neuronal function. Further in vivo investigations will be necessary to fully explore S1P_2_ as a reliable pharmacological target for treating CIPN.

## AUTHOR CONTRIBUTIONS

Brenda Wan Shing Lam designed and performed the experiments, and drafted the manuscript. Ping Xiang designed and performed the experiments. Boya Peng drafted the manuscript. Ling Jun Joshua Soon, Amelia Ting Yu Yam, Claudine Ming Hui Lim, and Yu Zheng performed the experiments. Long N. Nguyen supervised the study. Deron R. Herr conceived and supervised the study, and reviewed and revised the manuscript. Minh T. N. Le supervised the study, reviewed and revised the manuscript, and provided funding support.

## CONFLICT OF INTEREST STATEMENT

The authors do not declare any conflict of interest.
